# Can Urban-Rural Patterns of Hospital Selection Be Changed Using a Report Card Program? A Nationwide Observational Study

**DOI:** 10.3390/ijerph15091827

**Published:** 2018-08-24

**Authors:** Tsung-Hsien Yu, Nikolas Matthes, Chung-Jen Wei

**Affiliations:** 1 Department of Health Care Management, National Taipei University of Nursing and Health Sciences, Taipei 108, Taiwan; ericthyu@ntu.edu.tw; 2 Department of Health Policy and Management, Bloomberg School of Public Health, Johns Hopkins University, Baltimore, MD 21205, USA; nmatthe1@jhu.edu; 3 Department of Public Health, Fu-Jen Catholic University, Taipei 242, Taiwan

**Keywords:** report card, healthcare-seeking, difference, income, residential area

## Abstract

Background: Guiding patients to choose high-quality healthcare providers helps ensure that patients receive excellent care and helps reduce health disparities among patients of different socioeconomic backgrounds. The purpose of this study was to examine and compare the effect of implementing a report-card program on the patterns of hospital selection in patients from different socioeconomic subgroups. Patients undergoing total knee replacement (TKR) surgery were used as the sample population. Methods: A patient-level, retrospective, observational and cross-sectional study design was conducted. Taiwan National Health Insurance claims data were used and all patients in this database who had received TKR between April 2007–March 2008 (prior to report-card program implementation) and between April 2009–March 2010 (after program implementation) were included. Those patients who were under 18 years of age or who lacked area-of-residence or National Health Insurance premium information were excluded. Travelling distance to the hospital and level of hospital performance were used to evaluate the effect of the report-card program. Results: A total of 32,821 patients were included in this study. The results showed that patterns of hospital selection varied based on the socioeconomic characteristics of patients. In terms of travelling distance and hospital selection, the performance of urban and higher income patients was shorter and better, respectively, than their rural and lower-income peers both before and after report-card-program implementation. Moreover, although the results of multivariate analysis showed that the urban-rural difference in travelling distance enlarged (by 4.75 km) after implementation of the report-card program, this increase was shown to not be significantly related to this program. Furthermore, the results revealed that implementation of the report-card program did not significantly affect the urban-rural difference in terms of level of hospital performance. Conclusions: A successful report-card program should ensure that patients in all socioeconomic groups obtain comprehensive information. However, the results of this study indicate that those in higher socioeconomic subgroups attained more benefits from the program than their lower-subgroup peers. Ensuring that all have equal opportunity to access high-quality healthcare providers may therefore be the next issue that needs to be addressed and resolved.

## 1. Background

Reducing health disparities is a key priority of governments worldwide. Several studies have proposed that health disparities will not improve without first improving quality of care [1,2,3]. Moreover, the findings of several other studies support the validity of this proposition [4,5].

In addition to improving the quality of healthcare providers, guiding patients to choose high-quality healthcare providers is a strategy for helping patients receive excellent care [6,7]. Information asymmetry characterizes the healthcare industry. Report cards are one of the methods used to eliminate the gap between healthcare providers and patients. The idea behind report cards is simple: healthcare providers possess much better information than patients about the quality of their services. Lacking good information, patients may overestimate or underestimate quality, which may lead to their making inappropriate choices. Concurrently, healthcare providers may be tempted to behave opportunistically in order to take advantage of patient ignorance. These problems may, in theory, be alleviated through the use of report cards [8].

Report-card programs have now been implemented widely in advanced countries. In Taiwan, the Bureau of National Health Insurance launched a report-card program in April 2008. Initially, the program released only 10 hospital-level indicators, which were associated with diabetes mellitus, total knee replacement and myomectomy. Since then, 39 additional indicators have been released for 7 diseases, including peptic ulcer, asthma, acute myocardial infarction and sinusitis.

The qualitative impacts of report-card programs have been discussed fully during the past two decades. Most of the related studies have focused on the effect of these programs on improving the quality of received healthcare, with positive effects identified in a majority of these studies [9,10,11] and inconclusive findings reported in a few studies [12,13,14]. Additionally, several studies investigated report-card format preferences from different perspectives [15,16]. However, these studies did not adequately examine the effect on patient characteristics, especially socioeconomic level (e.g., residential area and income level). Moreover, as most studies were conducted in North America and Europe, the current body of evidence overlooks the experience in Asia. Therefore, the purpose of the present study is to examine the effects of a report-card program on patterns of hospital selection in patients of different characteristics. Total knee replacement (TKR) surgery patients were targeted as the sample population.

## 2. Methods

### 2.1. Study Design

A patient-level retrospective, observational and cross-sectional study design was adopted.

### 2.2. Ethical Statement

The Institutional Review Board of the National Taiwan University Hospital approved the protocol that was used in the present study (protocol #201512014W). The dataset that was used included secondary, de-identified data only.

### 2.3. Data Source

The Taiwan National Health Insurance Research Database (NHIRD) was used in the present study. The NHIRD, published by the Taiwan National Health Research Institute, is a comprehensive set of all of the original claims data and registration files for the beneficiaries of the National Health Insurance (NHI) program. The database includes information on the 23 million Taiwanese enrollees (approximately 98% of the population) in the NHI program. The NHIRD is a de-identified, secondary database containing patient-level demographics and administrative information. The data is publicly available for research purposes. 

### 2.4. Study Population and Exclusion Criteria

The present study included data from all of the individuals in the database who underwent TKR surgery (ICD-9-OP code: 81.54) between April 2007–March 2008 (prior to report card implementation) and between April 2009–March 2010 (after report card implementation) at any hospital in Taiwan. Patients who were under 18 years of age (n = 20) were excluded as well as patients whose residential area (n = 61) and income status could not be identified (n = 83). Finally, a total of 32,821 patients were included in data analysis. 

### 2.5. Definition of Variables

#### 2.5.1. Independent Variables: Patient’s Socioeconomic Characteristics

Residential area and income status data were used to represent the socioeconomic status of patients. Detailed information on these data items follows below.

##### Residential Area

Residential area is associated with level of urbanization. However, Taiwan’s NHI is an occupation-based social insurance scheme and the employees of large enterprises may be enrolled using the address of their company’s headquarters rather than their address of residence. Thus, residential area was defined in the present study as the area where the patient had the most outpatient and pharmacy visits.

The location of each clinic and pharmacy was recognized as either urban or rural according to the definition of urbanization published by Taiwan’s National Health Research Institutes (NHRI). The 365 townships in Taiwan were classified into 7 levels based on the following indicators: population density (people/km^2^), proportion of the population holding an undergraduate degree or above, proportion of the population above 65 years of age, proportion of the population employed in agriculture and the number of physicians per 100,000 people. The residential areas that were located in level 1 to 3 were categorized as urban and other areas were categorized as rural.

##### Income Status

The patient’s NHI premium (monthly fee) was used as a proxy for income status. Patients’ insurance identification records were used to distinguish low-income patients from other patients. As low-income patients accounted for 1% of the NHIRD, the top 1% of insured level (NTD 92,000/USD 3000) was used to define high-income patients. Lastly, the remaining patients were categorized into one of two levels: the middle-high income level (insured level is higher than NTD 28,000/USD 900) and the middle-low income level.

#### 2.5.2. Dependent Variables: Patterns of Hospital Selection

Travelling distance to the hospital and the performance level of the selected hospital were used as dependent variables. The details follow below.

##### Travelling Distance to Hospital

Travelling distance was defined as the distance between the center of the town where hospitals were located and where patients resided. Because identifying information for patients and hospitals are not available in the NHIRD, the home addresses of patients could not be retrieved. Therefore, the center of the town where hospitals were located and patients resided was the only retrievable information. After obtaining patient area-of-residence information, we used the Geographic Information System (ArcGIS for desktop, version 10.3, ESRI, Redlands, CA, USA) to calculate the Euclidean distance between the center of the town in which hospitals were located and patients resided. The result was used as the travelling distance value.

##### Performance Level of the Selected Hospital

In the present study, the k-means clustering algorithm was used to classify the performance level of the selected hospital. The k-means clustering algorithm is based on cluster analysis, is one type of data mining approach and is one of the most-used methods for partitioning clusters [17]. We have previously applied this approach in prior research work [4,5]. The hospital-level annual operation volume, risk-adjusted average length of stay and 30-day readmission rate during the previous year for each period was calculated, with this information used as the parameters for the k-means clustering algorithm, which subsequently classified the hospitals into two quality-based groups.

#### 2.5.3. Covariates

The covariates used in the present study were patient age, gender and comorbidity status. In addition, healthcare-provider characteristics such as surgeon age, surgeon seniority, surgeon gender, hospital ownership, hospital accreditation status, hospital teaching status and hospital geographic location were included in the descriptive and bivariate analysis.

### 2.6. Statistical Analysis

All statistical analyses were performed using SAS (version 9.4, SAS Institution Inc., Cary, NC, USA). In statistical testing, obtaining a two-sided *p*-value ≤ 0.05 was considered to be statistically significant. The distributional properties of continuous variables were expressed using mean ± standard deviation (SD), whereas categorical variables were represented using frequency and percentage. In the univariate analysis, the potential predictors of dependent variables were examined using a chi-square test or a two-sample *t*-test, as appropriate. Next, a multivariate analysis was conducted using multiple linear regression and multivariate logistic regression in order to estimate the discrete effects of the report-card program on the healthcare-seeking behavior of patients of different socioeconomic strata.

## 3. Results

A total of 32,821 patients who underwent TKR surgical procedures from a total of 910 physicians in a total of 245 hospitals were included in the present study. The descriptive analysis (see Table 1) showed that the mean age of patients was 70 years and that three-quarters of the patients were female. Around 80% patients lived in urban areas, 16% were of higher-income status and the average travelling distance was 18.91 km. In terms of comorbidities, the mean score of the Charlson comorbidity index (CCI) was 1.29 and the numbers of patients with diabetes mellitus (DM), congestive heart failure (CHF), obesity and renal failure and insufficiency (RF) were 7662 (23.3%), 1886 (5.8%), 11 (0.03%) and 782 (2.4%), respectively.

In terms of surgeon and hospital-related characteristics, the mean age of surgeons was 48.55 years and their average seniority was 8.09 years. Around 80% of the patients received their surgery from an orthopedic surgeon. Moreover, the data showed that patients were likely to receive their surgery at hospitals with a relatively high accreditation level, although the difference was slight and that the majority patients went to nonprofit (46.15%) and public (34.25%) hospitals. The hospital-level average operation volume, risk-adjusted length of stay and readmission rate in the previous year were 341.74%, 7.85% and 0.59%, respectively.

Table 1 also shows the differences pre- and post-report card program implementation. Patients who underwent TKR before program implementation were younger and had a higher percentage of CHF, lower percentage of DM and lower CCI scores. But, the percentage of living in urban areas was higher and the travelling distance was longer. Besides, patients who received surgery prior to the report-card program were attended by surgeons who were younger and had less seniority. However, the percentage of orthopedic surgeon was lower. As for hospital characteristics, patients who received their operation prior to program implementation were more likely to be treated at medical centers, regional hospitals and public and private hospitals, with post-program patients showing increased treatment at regional and nonprofit hospitals. Furthermore, the hospitals that performed the TKR operations had lower operation volumes and longer/higher average risk-adjusted length of stays and 30-day readmission rates, respectively, prior to program implementation than after program implementation. Finally, after k-mean clustering algorithm classification, the percentage of patients who received their surgery at an excellent-performance hospital prior to program implementation was lower than after program implementation (29.33% vs. 30.53%).

Table 2 compares rural and urban patients before and after implementation of the report-card program. The results reveal that certain characteristics, including patient age, status of DM and RF, CCI, income status, distance to hospital, age and seniority of the surgeon and ownership and accreditation level of the hospital, differed significantly between rural and urban-dwelling patients prior to program implementation. These differences were largely equalized after implementation. Of note, patterns of hospital selection differed between urban and rural patients, with urban patients associated with a shorter mean travelling distance to a hospital, a shorter mean travelling distance to an excellent-performance hospital and a higher percentage of surgeries in excellent-performance hospitals than rural patients. Besides, a stratified analysis showed that, before the report card program was implemented, rural and higher income patients traveled slightly longer than rural and lower income patients to reach excellent-performance hospitals (63.7 km vs. 60.9 km). However, the travelling distances for higher and lower income patients in urban areas were almost the same (16.1 km vs. 16.2 km). After the report-card-program, the travelling distances to excellent-performance hospitals decreased for all except urban and lower-income patients. Furthermore, although the difference in the travelling distances of rural and urban patients reduced, the gap between lower and higher income patients grew after the program was implemented. Thus, the extent of the reduction in travelling distance was greater among higher income patients than among lower income patients, although this difference did not reach statistical significance. In terms of the percentages of patients receiving surgery in excellent performance hospitals, the difference between higher and lower income patients living in urban settings did not change significantly after report-card program implementation (80.3:19.7 vs. 79.6:20.4) but did change significantly between the same patient subgroups living in rural settings (94.5:5.5 vs. 91.5:8.5).

Table 3 compares higher-income and lower-income patients before and after the implementation of the report-card program. Similar to the findings for area of residence, significant differences in covariates between higher and lower-income patients were observed. Moreover, patterns of hospital selection differed between higher and lower income patients, with higher income patients associated with a shorter mean travelling distance to a hospital, a shorter mean travelling distance to an excellent-performance hospital and a higher percentage of surgeries in excellent-performance hospitals than their lower income peers. Again, the stratified analysis demonstrated that the advantaged group received more benefits from the report-card program. 

Finally, Table 4 displays the results of multivariate analysis. With regard to travelling distance, after adjusting for covariates, the data showed that, while the travelling distances of higher and lower income patients were statistically similar (β = 1.3801, *p*-value = 0.1040), the areas of residence of these groups were statistically different (β = 22.676, *p*-value < 0.0001). The data further showed that the report-card program enlarged the urban-rural difference in travelling distance to 4.75 km (β = 4.7522, *p*-value < 0.0001) but did not statistically change the difference in travelling distance, between higher and lower-income patients (β = −0.2552, *p*-value = 0.8391). In terms of hospital performance, after adjusting for covariates, the results showed that, although disadvantaged (lower income or rural) patients had less opportunity to receive TKR surgery in excellent-performance hospitals (β = −0.0054, *p*-value = 0.0007; β = −0.1477, *p*-value < 0.0001) than their advantaged peers, implementation of the report-card program significantly increased the ratio (β = 0.0531, *p*-value = 0.006) of this group that received care in excellent-performance hospitals. Regarding the effect of the report-card program on the difference between advantaged and disadvantaged populations, this study found a slight but not statistically significant increase in this difference.

## 4. Discussion

The recent increase in consumer rights protection legislation and patient-rights activities reflect growing public awareness of health-related information [18]. Over the past decade, Western countries have invested the resources necessary to make the public aware of quality information related to the healthcare market. Information disclosure policies encourage hospitals to improve quality of service and to enhance healthcare-market-related efficiencies [19]. ‘Report cards’ provide a quality-focused scorecard for individual hospitals. The public may use these report cards to vet hospitals and select those that have been evaluated as offering higher quality healthcare and safeguarding the medical rights of patients [20]. Although previous studies have examined the effects of report-card programs, no study has yet examined the impact of various patient characteristics on the report card. The present study may not only be the first study to investigate the effects of report card implementation in Taiwan but may also be the first to discuss whether these effects vary based on patient characteristics.

The findings of the relevant studies in the literature are inconsistent, with some finding that report cards had no effect [21,22,23] and some finding either small overall effects [24] or effects only for younger and highly educated patients [22]. Even among those studies that found effects, several noted that these effects did not persist over time [25,26]. The present study found that implementing a report-card program reduced the travelling distance and also increased the percentage of patients who underwent surgery in excellent-performance hospitals. But we also found the difference between advantage and disadvantage group might be enlarged, after the program was implemented.

In addition, the results demonstrated that patients shifted to nonprofit and regional hospitals and that the number of patients who received TKR surgery at regional hospitals increased over time. This phenomenon may be explained by the structure of the healthcare system in Taiwan and Taiwanese culture. Of the approximately 500 hospitals in Taiwan during the study period, 23 were medical centers, 87 were regional hospital and the remainder were community hospitals. Taiwanese people tend to receive surgery in mid- and large-size hospitals. However, as it is not easy to schedule surgery at medical centers, patients most often opt to receive surgery in regional hospitals. Further, regional hospitals are more likely than community hospitals to retain orthopedic surgeons on staff. The above reasons might may explain the observed shift of patients to nonprofit and regional hospitals and the rise in the number of patients receiving TKR surgery at regional hospitals.

Nevertheless, it is still worth noting that the pre-/post-implementation difference in undergoing surgery in excellent performance hospital for higher income and urban patients was greater than for lower income and rural patients. Travelling distance is one of the factors that is considered by patients when seeking medical treatment. Most hospitals in Taiwan are located in urban areas. Hence, the data showing that urban-dwelling patients had shorter travelling distances than their rural-dwelling peers are not surprising. In addition, the data suggested that a higher percentage of lower income patients than higher income patients lived in rural areas, which may also help explain why lower income patients reported longer travelling distances. With regard to hospital selection, the findings of the present study showed that the percentage of selecting excellent-performance hospitals was higher for urban and higher income patients than for rural and lower income patients. Residential area and income level are commonly used indicators for defining socioeconomic status and the literature has positively correlated socioeconomic status with health literacy. Eliminating the gap in health literacy is one of the strategies currently being used to reduce surgical disparities in United States [27].

With regard to the impact of the report-card program in Taiwan, the present study found that the patterns of hospital selection had changed after program implementation. However, the difference between the advantaged and disadvantaged groups grew as well, with the advantaged group gaining more benefits than the disadvantaged group from the program. Thus, the question remains as to why the report card program had disparate effects on different population subgroups. Studies have shown that rural dwellers have worse health literacy [28] and less health-related internet use [29]. One study indicated that the reasons for report-card program ineffectiveness included a lack of public awareness of available information and a poor match between the content and clarity of available information and user needs [30]. Urban and higher income patients typically are better educated and have better resources (e.g., knowledge, skills, kinfolks and friends) for making informed decisions on where to seek optimal healthcare. This may help explain why urban and higher income patients realized relatively more benefits from the report-card program in the present study.

After resolving the need for high-quality, easily understood information, the next issue to be addressed may be to ensure that everyone has equal access to high quality care. Several studies have indicated that poor people are most likely to visit nearby health facilities [31]. Hospitals in Taiwan are not distributed equally. While the mean travelling distance for rural and lower-income patients was shorter after program implementation, these patients may still perceive travelling distance as their first priority in selecting a healthcare provider. There are several potential strategies for improving equal access for disadvantaged populations. The first is surgery centralization. The issue of centralizing or decentralizing healthcare services has been discussed for decades [32,33]. Although centralization may reduce accessibility, this problem may be alleviated through careful implementation procedures that include establishing appropriate, minimum service volumes and establishing referral centers in every region. However, hospital capacity/staff workloads are another issue that must be considered and appropriately addressed. The second potential strategy is subsidizing transportation and postoperative care for disadvantage populations. Studies indicate that poor people are more likely than their wealthier counterparts to visit geographically close health facilities [34]. As poorer patients are often cared for by family members, the expenses related to travelling and post-operative care may be another key issue that patients consider when selecting a hospital. Appropriate subsidies may improve this situation. Last but not least, improving quality continuously and reducing qualitative differences among healthcare providers remain necessary.

### Limitations

The present study had several limitations. Firstly, the observational approach taken disallow the inference of causal relationships from the results. Secondly, the estimations of travelling distance used in the present study may be inaccurate. As mentioned in the Method section, place-of-residence information for patients was not accessible from the NHIRD. Although the method that was used in the present study to estimate travelling distance was adopted from other published studies, the potential for bias and inaccuracies in these estimations cannot be determined. Finally, the results of this study should be interpreted carefully for avoiding ecological fallacy. 

## 5. Conclusions

The patterns of hospital selection were changed after implementation of the report card program. However, differences between urban/rural patients and higher/lower income patients persisted. Ensuring that everyone has the same opportunity to access high-quality healthcare providers may be the next issue that needs to be addressed and resolved.

## Figures and Tables

**Table 1:** Descriptive analysis.

	All (n = 32,821)	Pre (n = 15,208)	After (n = 17,613)	*p*-Value
Patient’s characteristics				
Age, mean (S.D)	70.09 (8.3)	69.98 (8.4)	70.18 (8.3)	0.0313 ^§^
Gender (Female), n (%)	24,685 (75.2)	11,471 (75.4)	13,214 (75.0)	0.3988 ^¶^
DM (Yes), n (%)	7662 (23.3)	3411 (22.4)	4251 (24.1)	0.0003 ^¶^
CHF (Yes), n (%)	1886 (5.8)	921 (6.1)	965 (5.5)	0.0251 ^¶^
Obesity (Yes), n (%)	11 (0.03)	7 (0.05)	4 (0.02)	0.2498 ^¶^
RF (Yes), n (%)	782 (2.4)	317 (2.1)	465 (2.6)	0.0010 ^¶^
Comorbidity Index, mean (S.D)	1.29 (1.1)	1.27 (1.1)	1.31 (1.1)	0.0007 ^§^
Residential area (urban), n (%)	25,539 (77.8)	11,942 (78.5)	13,597 (77.2)	0.0039 ^¶^
Income status (higher income), n (%)	5298 (16.1)	2410 (15.9)	2888 (16.4)	0.1768 ^¶^
Distance to hospital (km), mean (S.D)	18.9 (42.7)	19.5 (43.6)	18.4 (41.9)	0.0304 ^§^
Distance to excellent-performance hospital (km), mean (S.D)	23.8 (48.9)	24.3 (49.3)	23.3 (48.5)	0.3232 ^§^
Surgeon’s characteristics				
Surgeon’s age, mean (S.D)	48.55 (7.6)	48.13 (7.4)	48.90 (7.7)	<0.0001 ^§^
Surgeon’s gender (Male), n (%)	32,784 (99.9)	15,186 (99.9)	17,598 (99.9)	0.1994 ^¶^
Surgeon’s seniority, mean (S.D)	8.09 (6.0)	7.32 (6.1)	8.77 (5.8)	<0.0001 ^§^
Surgeon’s specialty (Orthopedics), n (%)	26,152 (79.7)	11,320 (74.4)	14,832 (84.2)	<0.0001 ^¶^
Hospital characteristics				
Hospital ownership				<0.0001 ^¶^
Public, n (%)	11,241 (34.3)	5273 (34.7)	5968 (33.9)	
Nonprofit, n (%)	15,148 (46.2)	6694 (44.0)	8454 (48.0)	
Private, n (%)	6432 (19.6)	3241 (21.3)	3191 (18.1)	
Hospital accreditation level				<0.0001 ^¶^
Medical center, n (%)	12,275 (37.4)	6019 (39.6)	6256 (35.5)	
Regional hospital, n (%)	10,962 (33.4)	4589 (30.2)	6373 (36.2)	
Community hospital, n (%)	9584 (29.2)	4600 (30.3)	4984 (28.3)	
Hospital-level volume, mean (S.D)	341.7 (285.3)	315.8 (264.8)	364.2 (300.1)	<0.0001 ^§^
Hospital-level LOS, mean (S.D)	7.9 (1.7)	8.0 (1.7)	7.7 (1.7)	<0.0001 ^§^
Hospital-level readmission (%), mean (S.D)	0.6 (1.1)	0.8 (1.3)	0.4 (0.8)	<0.0001 ^§^
Received surgery in excellent-performance hospital, n (%)	9849 (30.3)	4412 (29.0)	5437 (30.9)	0.0002 ^§^
Numbers of hospital	245	217	212	
Numbers of surgeon	910	730	792	

^¶^ χ^2^ test; ^§^ *t*-test; CHF: Congestive heart failure; DM: Diabetes Mellitus; RF: Renal failure and Renal insufficiency; LOS: Length of Stay.

**Table 2:** Before-after report-card program comparison: stratified by residential area.

	Before Report-Card			After Report-Card			Before-after Comparison	
	Rural (n = 3266)	Urban (n = 11,942)	*p*-Value	Rural (n = 4016)	Urban (n = 13,597)	*p*-Value	*p*-Value (Rural-Rural)	*p*-Value (Urban-Urban)
Patient’s characteristics								
Age, mean (S.D)	69.36 (7.8)	70.15 (8.5)	<0.0001 ^§^	69.84 (8.0)	70.28 (8.4)	0.0026 ^§^	0.0092 ^§^	0.2271 ^§^
Gender (Female), n (%)	2464 (75.4)	9007 (75.4)	0.9802 ^¶^	2975 (74.1)	10,239 (75.3)	0.1152 ^¶^	0.1826 ^¶^	0.8250 ^¶^
DM (Yes), n (%)	681 (20.9)	2730 (22.9)	0.0147 ^¶^	913 (22.7)	3338 (24.6)	0.0182 ^¶^	0.0533 ^¶^	0.0016 ^¶^
CHF (Yes), n (%)	188 (5.8)	733 (6.1)	0.4177 ^¶^	232 (5.8)	733 (5.4)	0.3449 ^¶^	0.9701 ^¶^	0.0104 ^¶^
Obesity (Yes), n (%)	3 (0.09)	4 (0.03)	0.1682 ^¶^	1 (0.02)	3 (0.02)	0.9165 ^¶^	0.2252 ^¶^	0.5819 ^¶^
RF (Yes), n (%)	50 (1.5)	267 (2.2)	0.0125 ^¶^	94 (2.3)	371 (2.7)	0.1779 ^¶^	0.0136 ^¶^	0.0118 ^¶^
Comorbidity Index, mean (S.D)	1.22 (1.1)	1.28 (1.7)	0.0027 ^§^	1.28 (1.0)	1.32 (1.1)	0.0366 ^§^	0.0181 ^§^	0.0080 ^§^
Income status (higher income), n (%)	216 (6.6)	2194 (18.4)	<0.0001 ^¶^	316 (7.9)	2572 (18.9)	<0.0001 ^¶^	0.0407 ^¶^	0.2657 ^¶^
Distance to hospital (km), mean (S.D)	41.09 (60.1)	13.55 (35.6)	<0.0001 ^§^	36.03 (56.1)	13.24 (35.0)	<0.0001 ^§^	0.0002 ^§^	0.4891 ^§^
Distance to excellent hospital (km), mean (S.D)	61.06 (73.5)	16.19 (37.5)	<0.0001 ^§^	54.54 (70.3)	15.94 (38.2)	<0.0001 ^§^	0.0537 ^§^	0.7698 ^§^
Lower income, mean (S.D)	60.90 (73.2)	16.23 (37.3)	<0.0001 ^§^	55.67 (70.7)	16.24 (38.3)	<0.0001 ^§^	0.1359 ^§^	0.9896 ^§^
Higher income, mean (S.D)	63.71 (79.8)	16.06 (38.2)	<0.0001 ^§^	42.26 (64.8)	14.80 (38.0)	<0.0001 ^§^	0.0991 ^§^	0.5089 ^§^
Surgeon’s characteristics								
Surgeon’s age, mean (S.D)	47.26 (7.3)	48.37 (7.5)	<0.0001 ^§^	47.69 (7.5)	49.26 (7.7)	<0.0001 ^§^	0.0157 ^§^	<0.0001 ^§^
Surgeon’s gender, n (%)	3261 (99.9)	11,925 (99.9)	0.8726 ^¶^	4010 (99.9)	13,588 (99.9)	0.1122 ^¶^	0.7580 ^¶^	0.0839 ^¶^
Surgeon’s seniority, mean (S.D)	6.79 (5.7)	7.47 (6.2)	<0.0001 ^§^	8.50 (5.5)	8.85 (5.9)	0.0006 ^§^	<0.0001 ^§^	<0.0001 ^§^
Surgeon’s specialty (Orthopedics), n (%)	2399 (73.5)	8921 (74.7)	0.1524 ^¶^	3476 (86.6)	11,356 (83.5)	<0.0001 ^¶^	<0.0001 ^¶^	<0.0001 ^¶^
Hospital’s characteristics								
Hospital ownership			<0.0001 ^¶^			<0.0001 ^¶^	<0.0001 ^¶^	<0.0001 ^¶^
Public, n (%)	857 (26.2)	4416 (37.0)		961 (23.9)	5007 (36.8)			
Nonprofit, n (%)	1838 (56.3)	4856 (40.7)		2540 (63.3)	5914 (43.5)			
Private, n (%)	571 (17.5)	2670 (22.4)		515 (12.8)	2676 (19.7)			
Hospital accreditation level			<0.0001 ^¶^			<0.0001 ^¶^	<0.0001 ^¶^	<0.0001 ^¶^
Medical center, n (%)	819 (25.1)	5200 (43.5)		862 (21.5)	5394 (39.7)			
Regional hospital, n (%)	1471 (45.0)	3118 (26.1)		2134 (53.1)	4239 (31.2)			
Community hospital, n (%)	976 (29.9)	3624 (30.4)		1020 (25.4)	3964 (29.2)			
Received surgery in excellent-performance hospital, n (%)	797 (24.4)	3615 (30.3)	<0.0001 ^¶^	1039 (25.9)	4398 (32.4)	<0.0001 ^¶^	0.1512 ^¶^	0.0004 ^¶^
Lower income, n (%)	753 (94.5)	2903 (80.3)	<0.0001 ^¶^	951(91.5)	3501 (79.6)	<0.0001 ^¶^	0.0153	0.4364 ^¶^
Higher income, n (%)	44 (5.5)	712 (19.7)		88 (8.5)	897 (20.4)			

^¶^ χ^2^ test; ^§^ *t*-test; CHF: Congestive heart failure; DM: Diabetes Mellitus; RF: Renal failure and Renal insufficiency.

**Table 3:** Before-after report-card program comparison: stratified by income status.

	Before Report Card			After Report Card			Before-after Comparison	
	Lower Income (n = 12,798)	Higher Income (n = 2410)	*p*-Value	Lower Income (n = 14,725)	Higher Income (n = 2888)	*p*-Value	*p*-Value (Lower-Lower)	*p*-Value (Higher-Higher)
Patient’s characteristics								
Age, mean (S.D)	70.1 (8.3)	69.22 (8.4)	<0.0001 ^§^	70.3 (8.4)	69.61 (8.2)	<0.0001 ^§^	0.0964 ^§^	0.0898 ^§^
Gender (Female), n (%)	9531 (74.5)	1940 (80.5)	<0.0001 ^¶^	10,976 (74.5)	2238 (77.5)	0.0008 ^¶^	0.8983 ^¶^	0.0076 ^¶^
DM (Yes), n (%)	2845 (22.2)	566 (23.4)	0.1753 ^¶^	3513 (23.9)	738 (25.6)	0.0514 ^¶^	0.0014 ^¶^	0.0818 ^¶^
CHF (Yes), n (%)	779 (6.1)	142 (5.9)	0.7131 ^¶^	825 (5.6)	140 (4.9)	0.1030 ^¶^	0.0872 ^¶^	0.0917 ^¶^
Obesity (Yes), n (%)	5 (0.04)	2 (0.08)	0.3565 ^¶^	4 (0.03)	0 (0.00)	0.3757 ^¶^	0.5859 ^¶^	0.1215 ^¶^
RF (Yes), n (%)	254 (2.0)	63 (2.6)	0.0472 ^¶^	387 (2.6)	78 (2.7)	0.8238 ^¶^	0.0004 ^¶^	0.8452 ^¶^
Comorbidity Index, mean (S.D)	1.27 (1.1)	1.27 (1.0)	0.9094 ^§^	1.31 (1.1)	1.31 (1.0)	0.9726 ^§^	0.0021 ^§^	0.1443 ^§^
Residential area (urban), n (%)	9478 (76.2)	2194 (91.0)	<0.0001 ^¶^	11,025 (74.9)	2572 (89.1)	<0.0001 ^¶^	0.0127 ^¶^	0.0170 ^¶^
Distance to hospital (km), mean (S.D)	20.26 (44.5)	15.22 (37.6)	<0.0001 ^§^	19.18 (42.6)	14.65 (37.9)	<0.0001 ^§^	0.0401 ^§^	0.5867 ^§^
Distance to excellent hospital (km), mean (S.D)	25.43 (50.4)	18.84 (43.2)	0.0002 ^§^	24.66 (49.8)	17.25 (41.8)	<0.0001 ^§^	0.4935 ^§^	0.4399 ^§^
Urban, mean (S.D)	16.23 (37.3)	16.06 (38.2)	0.9176 ^§^	16.24 (38.3)	14.80 (38.0)	0.3148 ^§^	0.9896 ^§^	0.5089 ^§^
Rural, mean (S.D)	60.90 (73.18)	63.71 (79.82)	0.8058 ^§^	55.67 (70.72)	42.26 (64.84)	0.0867 ^§^	0.1359 ^§^	0.0991 ^§^
Surgeon’s characteristics								
Surgeon’s age, mean (S.D)	48.00 (7.37)	48.82 (7.75)	<0.0001 ^§^	48.78 (7.69)	49.53 (7.81)	<0.0001 ^§^	<0.0001 ^§^	0.0011 ^§^
Surgeon’s gender (Male), n (%)	12,781 (99.88)	2405 (99.79)	0.2621 ^¶^	14,712 (99.91)	2886 (99.93)	0.7485 ^¶^	0.4526 ^¶^	0.1679 ^¶^
Surgeon’s seniority, mean (S.D)	7.37 (6.09)	7.07 (6.14)	0.0258 ^§^	8.79 (5.81)	8.68 (5.96)	0.3838 ^§^	<0.0001 ^§^	<0.0001 ^§^
Surgeon’s specialty (Orthopedics), n (%)	9588 (74.93)	1732 (71.87)	0.0016 ^¶^	12,398 (84.20)	2434 (84.28)	0.9111 ^¶^	<0.0001 ^¶^	<0.0001 ^¶^
Hospital’s characteristics								
Hospital ownership			0.4095 ^¶^			0.3593 ^¶^	<0.0001 ^¶^	0.0987 ^¶^
Public, n (%)	4438 (34.7)	835 (34.7)		4958 (33.7)	1010 (35.00)			
Nonprofit, n (%)	5610 (43.8)	1084 (45.0)		7099 (48.2)	1355 (46.9)			
Private, n (%)	2750 (21.5)	491 (20.4)		2668 (18.1)	523 (18.1)			
Hospital accreditation level			<0.0001 ^¶^			<0.0001 ^¶^	<0.0001 ^¶^	<0.0001 ^¶^
Medical center, n (%)	4940 (38.6)	1079 (44.8)		5102 (34.7)	1154 (40.0)			
Regional hospital, n (%)	3954 (30.9)	635 (26.4)		5415 (36.8)	598 (33.2)			
Community hospital, n (%)	3904 (30.5)	696 (28.9)		4208 (28.6)	766 (26.9)			
Received surgery in excellent-performance hospital, n (%)	3656 (28.56)	756 (31.4)	0.0054 ^¶^	4452 (30.2)	985 (34.1)	<0.0001 ^¶^	0.0025 ^¶^	0.0347 ^¶^
Urban, n (%)	2903 (79.4)	712 (94.2)	<0.0001 ^¶^	3501 (78.6)	897 (91.1)	<0.0001 ^¶^	0.4003 ^¶^	0.0150 ^¶^
Rural, n (%)	753 (20.6)	44 (5.8)		951 (21.4)	88 (8.9)			

^¶^ χ^2^ test; ^§^ *t*-test; CHF: Congestive heart failure; DM: Diabetes Mellitus; RF: Renal failure and Renal insufficiency.

**Table 4:** Results of multivariate analysis.

	Travelling Distance		Patterns of Hospital Choice	
	β (se)	*p*-Value	β (se)	*p*-Value
Income level (ref = Higher)	1.3801 (0.8487)	0.1040	−0.0554 (0.0163)	0.0007
Residential area (ref = urban)	22.6756 (0.7489)	<0.0001	−0.1477 (0.0153)	<0.0001
Report Card (ref = Before)	−0.5068 (1.1469)	0.6586	0.0531 (0.0193)	0.0060
Report card X residential area	4.7522 (1.1136)	<0.0001	0.0028 (0.0153)	0.8574
Report card X income level	−0.2552 (1.2567)	0.8391	0.0115 (0.0163)	0.4807

N = 32,821. All models were adjusted by patient’s gender, status of congestive heart failure, diabetes mellitus, renal failure and renal insufficiency, obesity and comorbidity Index; β: regression coefficient; s.e: standard error.
